# Caffeoylquinic Acids from the Aerial Parts of *Chrysanthemum coronarium* L.

**DOI:** 10.3390/plants6010010

**Published:** 2017-02-17

**Authors:** Chunpeng Wan, Shanshan Li, Lin Liu, Chuying Chen, Shuying Fan

**Affiliations:** 1 College of Agronomy, Jiangxi Agricultural University, Nanchang 330045, China; lemonwan@126.com (C.W.); liss0824@126.com (S.L.); linliu0960@126.com (L.L.); ccy0728@126.com (C.C.); 2 Collaborative Innovation Center of Post-Harvest Key Technology and Quality Safety of Fruits and Vegetables in Jiangxi Province, Jiangxi Agricultural University, Nanchang 330045, China

**Keywords:** *Chrysanthemum coronarium* L., aerial parts, caffeoylquinic acids

## Abstract

To elucidate the chemical compositions of the aerial parts of *Chrysanthemum coronarium* L., the ethanol extracts of *Ch. coronarium* L. were firstly isolated by the MCI-gel resin column. The caffeoylquinic acid-rich fractions were further purified by various chromatographic columns including silica gel, Sephadex LH-20, and semi-preparative HPLC to yield the compounds. The purified compounds were characterized by ^1^H-Nuclear Magnetic Resonance (^1^H-NMR), ^13^C-NMR, and high resolution electrospray ionisation mass spectral (HR-ESI-MS) spectroscopy. Seven caffeoylquinic acid (CQA) compounds were isolated from this plant. Their structures were clarified by spectrometric methods and identified as 3-*O*-caffeoylquinic acid (**1**), 5-*O*-caffeoylquinic acid (**2**), 4-*O*-caffeoylquinic acid (**3**), 3,4-di-*O*-caffeoylquinic acid (**4**), 1,5-di-*O*-caffeoylquinic acid (**5**), 3,5-di-*O*-caffeoylquinic acid (**6**), and 4,5-di-*O*-caffeoylquinic acid (**7**). Caffeoylquinic acids were the major constituents present in the aerial parts of *Ch. coronarium* L. All of the isolates except for compounds **2** and **6** were reported for the first time from this species. Moreover, compounds **3**–**5**, and **7** were identified from the *Chrysanthemum* genus for the first time.

## 1. Introduction

*Chrysanthemum coronarium* L., commonly known as “Tonghao”, is used as an edible vegetable and medicinal plant, and belongs to the genus of *Chrysanthemum* (Compositae) [1]. Many previous studies have reported the isolation, identification, and the biological activities of the plant. The results have shown that flavonoids [2], phenolic acids [3], sesquiterpene lactone [4], monoterpene [5], diterpene [6], glycosyldiglycerides [7], alkaloid [8], phytosterol [9], heterocyclic compounds [8], polyacetylenes [9], and essential oils [10,11] were the major chemical constituents present in the plant. *Ch. coronarium* L. has shown a variety of biological activities including the elimination of phlegm, plant allelopathy, nematicidal activity, cytotoxic activity, antioxidant and free radical scavenging, insect antifeedant, hepatic protection, and antimicrobial properties [1]. Flavonoids and phenolic acids are responsible for the plant allelopathy and antioxidant and free radical scavenging activities [3]; polyacetylenes and essential oil are responsible for the insect antifeedant [12] and antimicrobial activities [10]; and terpene, in particular sesquiterpene lactone, is responsible for the cytotoxic [13] and antimicrobial activities [6].

Flavonoids and polyphenols are the characteristic constituents of *Ch. coronarium* L., which are also the main bioactive constituents of the Compositae family [2,3]. Caffeoylquinic acids (CQAs) are cinnamate conjugates, which are biosynthesized through the phenylpropanoid pathway. These phenolic compounds are generally involved in plant disease-resistance responses to biotic or abiotic stress [14]. Preliminary studies on the chemical constituents of the aerial parts of *Ch. coronarium* L. indicated that the caffeoylquinic acids were the major components in the plant, however, only three CQA compounds were elucidated until now; 3,5-di-*O*-caffeoyl-4-succinylquinic acid, chlorogenic acid, and 3,5-di-*O*-caffeoylquinic acid [3].

Herein, the isolation and structure elucidation of caffeoylquinic acids (CQAs) from the aerial parts of *Ch. coronarium* was achieved.

## 2. Results

**Figure 1:**
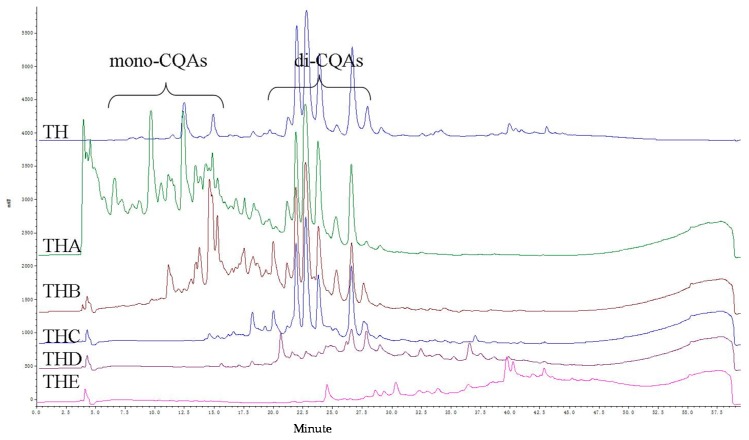
HPLC chromatogram of ethanol extract (TH) and MCI fractions (THA–THE) of *Ch. coronarium* L.

Caffeoylquinic acid derivatives showed typical UV spectra peaks at 327, 298 (sh), and 246 nm [15]. The HPLC profile of the ethanolic extract (TH) and of its five purified fractions (THA to THE) indicated that THA−THC were caffeoylquinic acid derivative-rich fractions (Figure 1). Many components in the THB fraction overlapped with the THA and THC fractions based on the HPLC profile. Thus, the THA and THC fractions were further isolated to yield the pure compounds.

**Figure 2:**
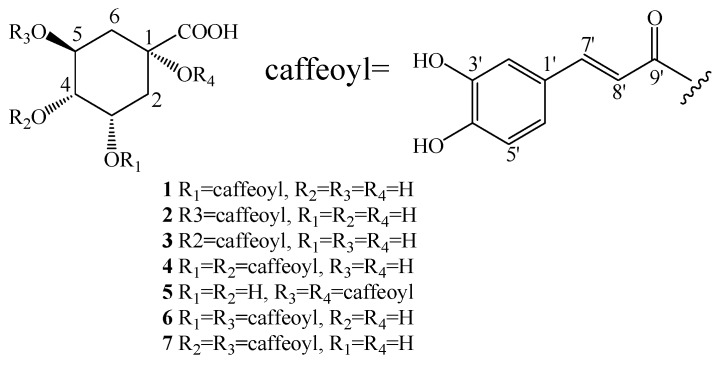
The chemical structures of the compounds **1**–**7** isolated from *Ch. coronarium* L.

The structures of the isolated caffeoylquinic acid derivatives were elucidated by HR-ESI-MS analysis, 1D-NMR data (Table 1 and Table 2), and comparison of these data with the literature. Compounds **1**–**7** (Figure 2) were identified as 3-*O*-caffeoylquinic acid (**1**) [16], 5-*O*-caffeoylquinic acid (**2**) [17], 4-*O*-caffeoylquinic acid (**3**) [16], 3,4-di-*O*-caffeoylquinic acid (**4**) [18,19], 1,5-di-*O*-caffeoylquinic acid (**5**) [20], 3,5-di-*O*-caffeoylquinic acid (**6**) [21], and 4,5-di-*O*-caffeoylquinic acid (**7**) [18,19]. Among them, compounds **1**, **3**–**5**, and **7** were isolated from this species for the first time. Moreover, compounds **3**–**5**, and **7** were reported from the genus *Chrysanthemum* for the first time.

## 3. Discussion

Compounds **1**−**3** were obtained as white power. The HR-ESI-MS yielded a quasi-molecular ion peak [M-H]^−^ at *m*/*z* 353.08. The UV spectrum showed λ_max_ at 328, 298 (shoulder), and 246 nm, which suggested that compounds **1**−**3** were single caffeoyl substituted quinic acid derivatives. The ^1^H-NMR spectrum (400 MHz, MeOH-*d*_4_) of compounds **1**–**3** showed caffeoyl signals at *δ* 7.56, 7.58, 7.63 (1H, d, *J* = 15.9 Hz, H-7′), 6.27, 6.30, 6.37 (1H, d, *J* = 15.9, H-8′), 7.04, 7.05, 7.06 (1H, H-2′), 6.94, 6.95, 6.97 (1H, H-6′), 6.78, 6.78, 6.78 (1H, H-5′), and quinic acid signals at [**1**: *δ* 5.36 (1H, brd, *J* = 2.9, H-3), 3.65 (1H, dd, *J* = 8.5, 2.8, H-4), 4.14 (1H, ddd, *J* = 8.5, 8.5, 3.6, H-5), 1.93–2.22 (4H, m, H-2, H-6); **2**: *δ* 4.18 (1H, brd, *J* = 2.9, H-3), 3.75 (1H, dd, *J* = 8.0, 2.3, H-4), 5.35 (1H, ddd, *J* = 8.0, 8.0, 3.5, H-5), 2.04–2.25 (4H, m, H-2, H-6); **3**: *δ* 4.29 (1H, brs, H-3), 4.80 (1H, dd, *J* = 9.0, 2.3, H-4), 4.27 (1H, ddd, *J* = 9.0, 9.0, 4.5, H-5), 1.98–2.23 (4H, m, H-2, H-6)]. The substituted position of caffeoyl can be determined by the analysis of the chemical shift and coupling constants of the oxygenated methine protons of the quinic acid core. Once the oxygenated methine of quinic acid was acylated by caffeoyl, the proton signal will shift downfield. The coupling constant of the downfield shifted proton was then applied to the acylation position. Generally, the H-3 signal has a small coupling constant and shows a brd or brs type peak, the H-4 signal showed a dd type peak with coupling constants at 8.0–9.0 Hz and 2.0–3.0 Hz, while the H-5 signal showed a ddd type peak with coupling constants at 8.0–9.0 Hz, 8.0–9.0 Hz, and 3.0–5.0 Hz. Based on these rules, the structures of compounds **1**–**3** were determined as depicted.

Compounds **4**–**7** were obtained as white powder. The ESI-MS yielded a quasi-molecular ion peak [M-H]^−^ at *m*/*z* 515.11, and the UV spectrum showed λ_max_ at 327, 298 (shoulder), and 245 nm, suggesting that compounds **4**–**7** were double caffeoyl substituted quinic acid derivatives. The ^1^H-NMR spectrum showed similar signal patterns to compounds **1**–**3**, but one more caffeoyl signal was observed. The ^1^H-NMR spectrum of compounds **4**–**7** showed two sets of caffeoyl signals [7.49–7.60 (each 1H, d, *J* = 15.9 Hz, H-7′ and H-7″), 6.97–7.05 (each 1H, H-2′ and H-2″), 6.85–6.96 (each 1H, H-6′ and H-6″), 6.71–6.78 (each 1H, H-5′ and H-5″), 6.17–6.33 (each 1H, d, *J* = 15.9 Hz, H-8′ and H-8″)], and quinic acid signals (see Table 1). Similar to compounds **1**–**3**, the acylation positions were determined by the chemical shift and coupling constants of the oxygenated methine protons of quinic acid. As only the H-5 signal of compound **5** was observed with a downfield shift, another substituted position was tentatively assigned to C-1 of quinic acid.

The chemical compositions of the plant are characterized by flavonoids and phenolic acids [22], which showed typical UV spectra based on the HPLC-DAD. In the current study, seven CQAs including three mono-CQAs and four di-CQAs were isolated and identified. Previously, two phenolic acids as plant growth inhibitors were isolated from *Ch. coronarium* L. and identified as isoferulic acid and methyl parahydroxybenzoats [12]. Ferulic acid methyl ester was also detected in *Ch. coronarium* L., which showed low-density lipoprotein (LDL) oxidation inhibited activity [8]. Only three quinic acid derivatives, namely, chlorogenic acid (5-*O*-caffeoylquinic acid, **2**), 3,5-di-*O*-caffeoylquinic acid (**6**), and 3,5-di-*O*-caffeoyl-4-succinylquinic acid were detected by the HPLC method [3]. This is the first report of the isolation of CQAs except for compounds **2** and **6**, while compounds **3**–**5**, and **7** were identified from the Chrysanthemum genus for the first time. Additionally, the di-CQAs are the major phenolic acid constituents present in this plant.

## 4. Materials and Methods

### 4.1. Plant Material

The aerial parts of *Ch. coronarium* L. were purchased from a local market, Nanchang City, Jiangxi Province, China, and identified by Prof. Shuying Fan (College of Agronomy, Jiangxi Agricultural University, Nanchang, China). A voucher specimen (TH-2015041) has been deposited in the Department of Horticulture, College of Agronomy, Jiangxi Agricultural University (Nanchang, Jiangxi, China).

### 4.2. Equipment and Reagents

^1^H and ^13^C**-**NMR were detected on a Varian 400 MHz spectrometer in CD_3_OD with Tetramethylsilane (TMS) as the internal standard. HR-ESI-MS data were obtained on a 6538 Ultra High Definition (UHD) Accurate-Mass Q-TOF LC/MS system (Agilent, Santa Clara, CA, USA). High performance liquid chromatography (HPLC) was performed on a Hitachi Elite Chromaster system including a 5110 pump, 5210 autosampler, 5310 column oven, a 5430 diode array detector, and operated by EZChrom Elite software. Luna C18 (2) column (5 µm, 4.6 × 250 mm) for analysis and Luna C18 (2) column (10 µm, 10 × 250 mm) for HPLC preparation were purchased from Phenomenex Inc (Torrance, CA, USA). The HPLC grade solvents were purchased from Sigma (Sigma, St. Louis, MO, USA). All analytical solvents were purchased from Tansoole (Shanghai, China). Silica gel (250 mesh; Qingdao Haiyang Chemical Co., LTD, Qingdao, China) was used as normal phase, whereas YMC Pack ODS-A (50 µm; YMC) was used as reversed phase column material. MCI gel CHP20P (75–150 µm; Mitsubishi Chemical Corp, Tsukuba, Japan) and Sephadex LH-20 (GE Healthcare, Uppsala, Sweden) were also used for column chromatography.

### 4.3. Extraction and Chromatography

The fresh aerial parts of *Ch. coronarium* L. (20 kg) were dried in air, yielding a crude dry material which amounted to about 2.2 kg. The dried material (2.0 kg) was ground and extracted using an ultrasonic-assisted method with 95% ethanol (3 × 50 L) at 45 °C for 2 h. The dried ethanol extract (TH, 118.5 g) was subjected to MCI gel column chromatography (4.0 × 25 cm), eluted with water, 10% methanol, 30% methanol, 50% methanol, 70% methanol, and 90% methanol, respectively (each, 2.0 L). Lastly, the MCI gel column was washed with acetone. Six fractions were yielded (THA–THF).

### 4.4. Purification of the Caffeoylquinic Acid Derivatives

The THA fraction (13.2 g) was subjected to ODS C18 column chromatography (3.0 × 25 cm) eluting with 5% methanol, 15% methanol, 25% methanol, 35% methanol, and 50% methanol (each 1.0 L), respectively. Five fractions (THA-**1**–THA-**5**) were obtained after being pooled according to their HPLC profiles.

Fraction THA-3 (3.3 g) was further subjected to Sephadex LH-20 (2.0 × 100 cm) elution with MeOH to furnish fractions THA-3A–3D. Fraction THA-3C was purified by semi-preparative HPLC (10 µm, 10 × 250 mm), eluting with MeOH-H_2_O (0–23 min: 18:82 to 45:55; *v*/*v*, 3 mL/min) and yielding compounds **1** (12.5 mg), **2** (26.8 mg), and **3** (14.5 mg).

**Figure 3:**
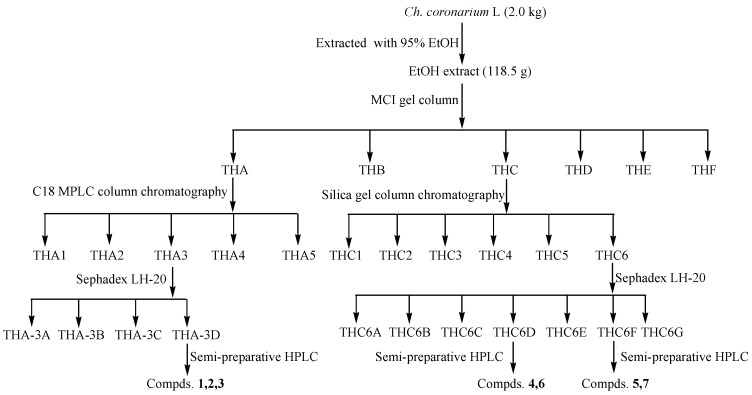
The procedure for the extraction and isolation of compounds from *Ch. coronarium* L.

The THC fraction (6.57 g) was subjected to silica gel chromatography (4.0 × 26 cm) using CHCl_3_-MeOH (100:1 to 2:1, *v*/*v*) for elution to yield six fractions THC-**1**–**6** according to their TLC profiles. THC-**6** (1.2 g) was further subjected to Sephadex LH-20 (2.0 × 100 cm) elution with MeOH to furnish fractions THC-6A–6G. THC-6D was purified by semi-preparative HPLC (10 µm, 10 × 250 mm), eluting with an isocratic elution of MeOH-H_2_O (34:66; *v*/*v*, 3 mL/min) yielding compounds **4** (8.5 mg) and **6** (9.8 mg). THC-6F was purified by semi-preparative HPLC (10 µm, 10 × 250 mm), eluting with an isocratic elution of MeOH-H_2_O(35:65; *v*/*v*, 3 mL/min) yielding compounds **5** (13.5 mg) and **7** (14.6 mg) (Figure 3).

## 5. Conclusions

Caffeoylquinic acids (CQAs) were the major phenolic constituents present in the aerial parts of *Ch. coronarium* L. Seven CQAs including three mono-CQAs and four di-CQAs were isolated from this plant. They were 3-*O*-caffeoylquinic acid (**1**), 5-*O*-caffeoylquinic acid (**2**), 4-*O*-caffeoylquinic acid (**3**), 3,4-di-*O*-caffeoylquinic acid (**4**), 1,5-di-*O*-caffeoylquinic acid (**5**), 3,5-di-*O*-caffeoylquinic acid (**6**), and 4,5-di-*O*-caffeoylquinic acid (**7**), respectively. All of the isolates except for **2** and **6** were isolated from this species for the first time. Moreover, compounds **3**–**5**, and **7** were identified from the Chrysanthemum genus for the first time.

## Figures and Tables

**Table 1:** ^1^H-NMR (^1^H-Nuclear Magnetic Resonance, 400 MHz, MeOH-*d*_4_) characteristics of the caffeoylquinic acid derivatives **1**–**7** isolated from aerial parts of *Ch. coronarium* L.

No.	1	2	3	4	5	6	7
	*δ*H (J Hz)	*δ*H (J Hz)	*δ*H (J Hz)	*δ*H (J Hz)	*δ*H (J Hz)	*δ*H (J Hz)	*δ*H (J Hz)
2	1.93–2.22 (2H, m)	2.04–2.25 (2H, m)	1.98–2.23 (2H, m)	2.07–2.35 (2H, m)	2.54 (1H, dd, 3.5, 10.2) 2.43 (1H, m)	2.12–2.32 (2H, m)	2.14–2.30 (2H, m)
3	5.36 (1H, brd, 2.9)	4.18 (1H, brd, 2.9)	4.29 (1H, brs)	5.61 (1H, brd, 3.4)	4.28 (1H, brd, 3.5)	5.36 (1H, brd, 5.6)	4.40 (1H, brs)
4	3.65 (1H, dd, 2.8, 8.5)	3.75 (1H, dd, 2.3, 8.0)	4.80 (1H, dd, 2.3, 9.0)	4.96 (1H, dd, 3.1, 9.4)	3.76 (1H, dd, 3.0, 8.1)	3.96 (1H, dd, 3.2, 7.3)	5.10 (1H, d, 8.5)
5	4.14 (1H, ddd, 3.6, 8.5, 8.5)	5.35 (1H, ddd, 3.5, 8.0, 8.0)	4.27 (1H, ddd, 9.0, 9.0, 4.5)	4.35 (1H, ddd, 4.2, 9.4, 9.4)	5.37 (1H, ddd, 3.7, 8.1, 8.1)	5.42 (1H, ddd, 3.2, 7.3, 7.3)	5.63 (1H, ddd, 4.2, 8.5, 8.5)
6	1.93–2.22 (2H, m)	2.04–2.25 (2H, m)	1.98–2.23 (2H, m)	2.07–2.35 (2H, m)	2.05 (1H, dd, 11.1, 13.8) 2.43 (1H, m)	2.12–2.32 (2H, m)	2.14–2.30 (2H, m)
2′/2″	7.04 (1H, d, 1.4)	7.05 (1H, brs)	7.06 (1H, d, 1.4)	7.02/7.00 (each 1H, d, 1.8)	7.04 (each 1H, brs)	7.05 (each 1H, s)	7.00/6.97 (each 1H, s)
5′/5″	6.78 (1H, d, 8.0)	6.78 (1H, d, 8.0)	6.78 (1H, d, 8.0)	6.76/6.72 (each 1H, d, 7.9)	6.78/6.76 (each 1H, d, 8.1)	6.77/6.75 (each 1H, d, 8.1)	6.73/6.71 (each 1H, d, 8.1)
6′/6″	6.94 (1H, dd, 1.4, 8.0)	6.95 (1H, d, 8.0)	6.97 (1H, dd, 1.4, 8.0)	6.90/6.85 (each 1H, dd, 1.8, 7.9)	6.96/6.94 (each 1H, d, 8.1)	6.96/6.94 (each 1H, m)	6.89/6.87 (each 1H, d, 8.1)
7′/7″	7.58 (1H, d, 15.9)	7.56 (1H, d, 15.9)	7.63 (1H, d, 15.9)	7.57/7.52 (each 1H, d, 15.9)	7.58/7.55 (each 1H, d, 15.9)	7.60/7.56 (each 1H, d, 15.9)	7.57/7.49 (each 1H, d, 15.9)
8′/8″	6.30 (1H, d, 15.9)	6.27 (1H, d, 15.9)	6.37 (1H, d, 15.9)	6.27/6.23 (each 1H, d, 15.9)	6.27/6.24 (each 1H, d, 15.9)	6.33/6.24 (each 1H, d, 15.9)	6.26/6.17 (each 1H, d, 15.9)

**Table 2:** ^13^C-NMR Data for Compounds **2**, **5**–**6** (100 MHz, MeOH-*d*_4_).

No.	Compounds		
	2	5	6
1	74.7	80.9	73.3
2	36.8	35.7	34.6
3	69.9	69.4	71.1
4	72.1	72.8	69.2
5	70.5	71.6	70.6
6	37.4	36.9	36.2
7	175.6	174.8	175.9
1′	126.4	127.8/127.8	126.5/126.4
2′	113.8	115.3/115.3	114.2/113.9
3′	145.3	147.6/147.4	145.3/145.3
4′	148.1	149.7/149.7	148.1/148.0
5′	115.1	116.5/116.5	115.1/115.1
6′	121.6	123.1/123.1	121.6/121.6
7′	145.7	147.4/146.9	145.9/145.6
8′	113.8	115.2/115.1	113.7/113.7
9′	167.3	168.7/168.0	167.4/167.0
